# A Cluster Randomized Trial to Evaluate a Health Education Programme “Living with Sun at School”

**DOI:** 10.3390/ijerph9072345

**Published:** 2012-07-02

**Authors:** Hélène Sancho-Garnier, Bruno Pereira, Pierre Césarini

**Affiliations:** 1 Centre Régional de Lutte Contre le Cancer Val d’Aurelle, Epidaure Prévention Dépt., 208 Rue des Apothicaires, Montpellier 34298, France; 2 Centre Hospitalier Universitaire, Direction de le Recherche Clinique, Biostatistics Unit, Clermont-Ferrand 63000, France; Email: bpereira@chu-clermontferrand.fr; 3 NGO: Sécurité solaire, Paris 75018, France; Email: p.cesarini@soleil.info

**Keywords:** sun-exposure, health education, school, knowledge, behaviour, cluster randomized trial

## Abstract

Over-exposure to sunlight increases the risk of skin cancers, particularly when exposure occurs during childhood. School teachers can play an active role in providing an education programme that can help prevent this. “Living with the Sun,” (LWS) is a sun safety education program for school children based on a handy guide for classroom activities designed to improve children’s knowledge, but moreover to positively modify their sun safety attitudes and behaviours. The goal of our study was to determine the effectiveness of this programme by examining children’s knowledge, attitude and sun exposure behaviours prior to and after the completion of the programme. We carried out a cluster randomised trial in which the classes were randomly assigned to one of two groups; one using the LWS programme and another that didn’t, serving as the control. Data was collected before completion of the programme and an additional three times in the year after completion. The 70 participating classes (1,365 schoolchildren) were distributed throughout France. Statistical analysis confirmed that knowledge of sun risk increased significantly in the LWS classes (*p* < 0.001). Both groups positively changed their attitudes when considering the best sun protection, but the LWS group proved to consistently be more convinced (*p* = 0.04). After the summer holidays, differences between the two groups decreased throughout the year but stayed globally significant. We also observed some significant behaviour modification during the holidays. For instance, the LWS group applied sunscreen more frequently than the control group, and were more likely to wear a hat (72% *versus* 59%) and use a sun umbrella on the beach (75% *versus* 64%).

## 1. Introduction

It has been clearly demonstrated that overexposure to sunlight increases the risk of skin cancers [1], cataracts [2], and age-related macular degeneration [3], particularly when exposure occurs during childhood [4]. Frequency of skin cancers has been increasing steadily worldwide for more than 30 years, and their prevention has become a public health priority in many countries where the population has mainly a white skin phenotype [5]. In France, there are approximately 80,000 new skin cancer diagnoses each year, of which nearly 10,000 are diagnosed as melanoma (Adjusted Standardized Rates World (asrw): 9.7/100,000 in men and 10.7/100,000 in women) [6]. Time trends in incidence for melanoma have increased since 1980 at +4.5% each year in men and +3.4% each year in women. However, since 2000, this tendency has slowed in both genders (respectively, 0.8%/year and 0.5%/year). The mortality (ASRW) time trends for melanoma diagnoses are 1.7 in men and 1.1 in females, and increased in men between 1980 and 2005, but presently are slowing. In women, time trends have decreased since the beginning of the 21st century [6].

Sun exposure should be modifiable through behavioural interventions such as avoiding midday sun, applying sunscreen, and by wearing protective clothing, like hats and sunglasses, *etc*. [7]. Such healthy behaviours have to be applied by the whole population, but particularly by young children. Despite this knowledge, to date, there is insufficient evidence that counselling by clinicians and parents alone has been really efficient [7,8]. Consequently, school teachers could be complementary, or even act as a better conduit for providing prevention advice and education [9]. Teachers spend a lot of time with children and better know how to increase their knowledge and modify behaviour through pedagogic models. In addition, existing school curricula in science, math, geometry and language can all be used to address the key elements of sun prevention. Furthermore, health education is already generally included in the school programmes. A limited number of Randomized Intervention trials (RIT) dealing with school-based interventions promoting sun-protective behaviours are reported in international literature. Among the published trials a great diversity of actions and methods have been used, but nearly all have the same final objective to increase sun protective behaviour [10,11,12,13,14,15,16,17,18,19,20,21,22]. Some other trials are more specifically dedicated to reduce the number of melanocytic nevi [23,24,25,26]. The criteria used to evaluate the results are dependent upon the type of actions. For example, if the increase in knowledge is generally well standardized and easy to measure, the modifications of attitude and behaviour are not, and may lead to heterogeneity and interpretation biases. The majority of the studies observe, as a consequence of their intervention, a global improvement in knowledge, but very few report a persistent modification in sun exposure attitude and behaviours [7,12,13,17] even though some paradoxical results are observed, like an increase of sun exposure after the intervention [10]. Furthermore, other determinants of success, like age and phenotypes of the targeted population, the duration of the intervention, the tools used and the end points of the evaluation, influence the results [13,27]. Finally, discrepancies between the results can also be explained by either a low statistical power or an inadequate statistical analysis of the cluster design. When the curriculum, “Living With the Sun”, was proposed for promotion in French primary schools, we considered that there was still room to undertake a new study taking into account the aforementioned problems.

“Living with the Sun” (LWS) is a transverse and multidisciplinary sun safety education guide for teachers. It was created in 2006 by an astrophysicist, a communications specialist and a teacher trainer and edited by the nongovernmental organization “Sécurité Solaire” [28]. It called for teachers to lead 10 workshops for 9- to 12-year old pupils during the last three months of the school year (from April to June in the northern hemisphere). The teaching guide contained practical and entertaining classroom activities designed to potentially improve children’s scientific knowledge and to positively modify their sun safety attitudes and behaviours. A first evaluation was performed in a limited area, including 282 children. The programme showed a beneficial effect in increasing children’s knowledge for a few months, but no long term difference concerning their sun protection habits [29]. Before disseminating the curriculum throughout France, a new, right comparative methodology was needed for the evaluation of the curriculum. Our goal was to determine the effectiveness of this preventive programme.

## 2. Methods

### 2.1. The Teaching Guide LWS: Composition and Use

This guide contains practical classroom work and activities designed to increase children’s scientific knowledge of the sun, its characteristics and activities in relation to life on the earth. For children, it helps them to understand how the sun works and what the benefits and risks of exposure to the sun are, so they can modify their own behaviours. The ten workshops, delivered during a three-month period, covered four topics: The effect of sun exposure on the body; the different skin types and their sensitivity to sunlight [30]; the determinants of variations in the UV intensity; and sun protection strategies. The specific concept of the LWS programme is to increase students’ competencies when dealing with the sun, not simply to enhance their academic knowledge. The investigative approach, which is at the heart of the programme, allows children to record observations of their physical environment, so they can raise questions and find answers to those questions through guided experiments in the programme. The hypothesis of this study is that an increase in children’s practical competency, beyond simple knowledge, could have a greater effect in changing their sun exposure behaviours.

To optimize the use of teaching time, proposed lessons corresponded with the official primary school curriculum in the sciences, geography, mathematics, and language. Enhancing the programme’s progress, different classes worked together on related projects and activities. For example, some groups created public information announcements to be displayed in schools throughout the district, others wrote petitions to local politicians about the lack of shade in local courtyards or at swimming pools. Another goal of this programme was that the children’s efforts would receive attention in the local press. Other advantages of this programme include that it targets the right age group to effect and modify behaviours for lasting change, by using a pragmatic, interactive and entertaining methodology to increase children’s self-esteem and knowledge about the sun and its relation to their health [31,32,33]. In addition, it is delivered by professional pedagogues in a group environment favouring peer effect [21,34]. Beginning with observation raising questions, the children find answers to their questions using invention, criticism, experimentation and evaluation. By engaging the student’s perception of his or her own ability to solve problems and to understand how the components of such problems work, this curriculum can help children realize the risks of sun exposure and empower them to modify their own behaviour for their health [35,36].

### 2.2. The Evaluation

#### 2.2.1. The Experimental Design

From April 2007 to December 2007 we carried out a cluster randomised trial [37] in which classes, rather than individuals, were stratified by regions and randomly assigned to one of two groups: One, using the LWS programme and one not, serving as control group. Cluster randomised trials are valuable in the evaluation of interventions with groups like school classes, families and villages. In such a trial, relationships between individuals in the group (because they have some similarities and influences on each other), have to be examined and eventually controlled for [38]. To avoid contamination bias, when randomized classes were found to belong to the same school they were considered as one class (this happened in only three schools).

#### 2.2.2. Data Collection and Measures

Data regarding the children’s knowledge, attitudes and behaviour were collected in 70 classes using the same questionnaires, paired child by child, at different times: Before intervention (T0), then after completion of the programme (T1), in 42 school classes, two months after the summer holidays (T2) and in 33 classes one year later (T3). Specific questions on behaviour were added at T2. All the questionnaires were filled out at school and were supervised by teachers who were trained before beginning the study. The questionnaires were composed of four parts: General and skin-type characteristics; knowledge of the sun; attitude concerning sun prevention; and sun behaviours. In the absence of already standardized validated scales [11], we used our own questionnaire already tested in previous studies. This questionnaire was created using excerpts from the literature, combined with our experiences in previous studies [7,39,40,41], and tested on a sample of seven classes (122 children). We analysed the internal validity as well as the comprehension, acceptability and reproducibility of the questions.

The questions were mostly binary; some were qualitative and codified afterward by a blind worker (not aware of the cluster type it comes from). Other measures were done using arrows linking items, or showing an answer). The order of the questions was not the same at the various times of the evaluation to avoid memorizing bias.

#### 2.2.3. Statistical Analysis

The initial participating classes (31 in the control group and 39 in the experimental one, or the LWS group), consisted of 1,365 school children with a mean age of 9.9 years and were distributed throughout France. This number was calculated to obtain a power of 90% (1-β) to detect a 10% difference at T1 (Standard Deviation (SD) = 30%) between the two groups in the two major criteria (global knowledge as percentage of good answers and global sun-behaviour during holidays), with an error risk α of 5% (two-sided test), and taking into account an intra-cluster coefficient equal to 0.10 with a mean cluster size of 25 children.

Because subjects were randomized by clusters, the classes in this case, techniques appropriate for clustered data were used. All analyses accounted for the correlation of measurements within classes. We summarized children and the schools characteristics overall and for each study arm. We tested the effectiveness of the randomization by comparing children and class characteristics across arms using practice-adjusted chi-square and t tests.

To compare the two groups, statistical analysis was conducted with mixed effects regression extensions (GLMM), accounting for dependencies among cluster members, adjusted on significant (*p* ≤ 0.05) co-variates at T0 and pre-test values [42]. Intraclass correlation coefficients from these models have been estimated. Bonferroni’s correction and false discovery rate, as introduced by Benjamini and Hochberg, were used when assessing the significance of multiple regression coefficients/correlations [43].

We performed a secondary analysis using all observations, including children missing baseline or follow-up measures. We estimated a repeated measures model for each outcome and tested the interaction between measurement periods and intervention group assignments [44]. These were linear four-level hierarchical models with observations nested within children, within clustered classes, and schools. In order to assess the problem caused by missing data (classes and children), we used the estimation methods developed by Verbeke and Molenberg [45].

All statistical analyses were performed using Stata 9 (Stata Corporation, College Station, TX, USA).The data was anonymous and, following French legislation in 2006, the file was registered at “Comité National Informatique et Liberté” (N° 1156768 on 20/03/2006). The information and the parental waivers were completed and obtained by participating school teachers.

## 3. Results and Discussion

### 3.1. Characteristics of the Children by Groups

As described in Table 1, there was no statistical difference observed concerning age, gender and photo-types or basic knowledge of sun risks. There were some differences observed concerning their outside activities: The control group practiced more often walking or cycling (*p* = 0.03) and swimming (*p* = 0.008). These parameters were used as adjustment covariates in the following analysis.

### 3.2. Modification of Knowledge between T0 and T1

The questionnaire about the children’s knowledge was composed of 47 questions, classified under 13 headings (Table 2).

**Table 1:** Characteristics of the two groups (LWS programme and control).

Characteristics (% item)		LWS group (798)	Control group (567)
Mean age		9.93 (SD * = 0.6)	9.87 (SD * = 0.8)
Gender	% girls	45.8	46.1
Eye colour:	% fair	34.3	35.5
Hair colour	% fair	49.5	52
Skin type I + II + III **	%	57.7	58.7
Freckles	% no	70.8	70.2
Easy sunburn	% yes	30.5	36

* SD: Standard deviation; ** from Fitzpatrick classification [30].

**Table 2:** Comparison of children’s knowledge about the sun in the two groups of classes at T0 and T1 by questionnaire headings (% of good answers).

Questionnaire Headings	T0		T1	
	CTRL ^1^	LWS ^2^	CTRL	LWS
Does the sun emit visible/invisible rays or gas to the earth?	57.7	58.8	58.7	63.8 ^3^
What rays produced by the sun can our eye see UV, IR, or white light?	65.9	55.6 ^3^	62.4	73.4 ^3^
Sun and earth trajectories	63.4	63.4	66.7	74.5 ^3^
The time of the day the sun has maximum intensity	28.4	33.4	29.0	49.4 ^3^
Risk for skin and eyes	62.8	61.5	62.2	78.2 ^3^
What are the good health effects of the sun?	62.0	63.4	65.4	79.4 ^3^
Sun exposure risks?	56.8	61.6	70.2	88.3 ^3^
Which clothes are more protective?	49.3	50.8	48.9	64.6 ^3^
Other types of protection?	74.3	73.5	80.4	88.2 ^3^
About sunscreen use	43.8	39.3	45.3	45.6
Use of protection index on sunscreen	59.5	60.2	61.6	70.1 ^3^
What to do in case of insolation?	75.2	75.4	79.5	82.4
True or false information	56.2	56.1	60.9	73.3 ^3^

^1^ Control classes; ^2^ Experimental classes; ^3^*p* ≤ 0.02.

At T0, out of 47 questions, the control group had better knowledge than the LWS group only twice (*p* ≤ 0.05); no other significant differences were observed. When pooling the questions by headings (Table 2), there is only one heading where the score differs at T0 (*p* = 0.02): The control group knew better what the eye can see (ultraviolet, infrared, white light). These parameters were used as adjustment covariates in the following analysis.

At T1, the gap in knowledge increased between the two groups in favour of the LWS group, which gives more frequently (*p* < 0.05) a right answer in 31 of the 47 questions.

The global score at T0 (% of good answers) for the 47 questions was 59.5% (SD = 11.4%) in the control group and 59.2% (SD = 9.9%) in the experimental group (*p* = 0.84). At T1 (*p* = 0.0001), it becomes 62.7% (SD = 10.7%) and 73% (SD = 10.4%), respectively.

**Figure 1:**
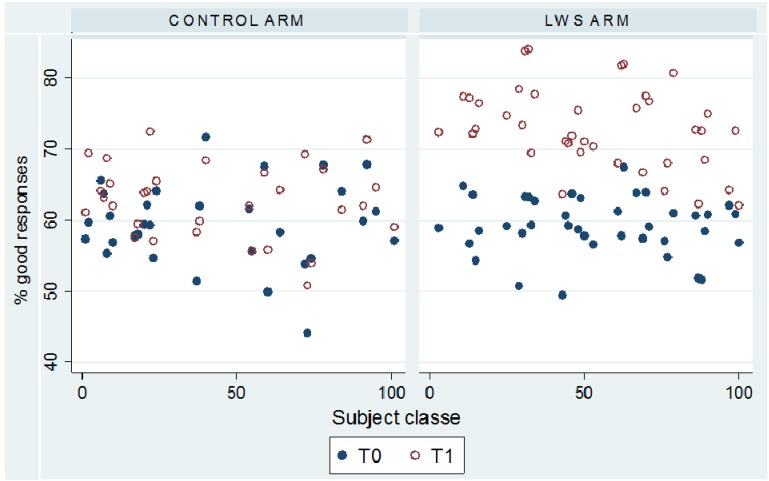
Evolution of knowledge rates within the various classes by intervention group.

Figure 1 illustrates the evolution of the knowledge in the 70 classes between T0 and T1. The regression model shows that children’s skin colour and predisposition to sunburn (Fitzpatrick classification) also affected the knowledge scores (*p* < 0.05). The lower the children’s skin type, the better their knowledge was of the sun’s activities. On the contrary, the children who answered that they have a tendency to sunburn had lesser knowledge in general. 

### 3.3. Modification of Attitude between T0 and T1

This section of the questionnaire contained 16 items in four headings (Table 3). At T0 we did not observe any difference between the two groups. Both believe that being tan looks better (47%); that it is necessary to protect themselves from the sun, particularly when they are practicing sports outside (72.5%) and on the beach (86.5%); they used sunscreen mostly to avoid sunburn (57%); and to be protected, they think it’s better to use all types of protection together (47.5%). However, at T1, in five of the 16 items, we observed a significant difference: The children from LWS classes think more often that to be tan (*i.e.*, to have a darker skin, as a consequence of a higher production of eumelanins) protects more from sunburn (*p* = 0.004) (48.6% *versus* 35.4%, respectively). In addition, they believe it is necessary to protect themselves when walking or being in the mountains (respectively, *p* = 0.03 and *p* < 0.000) and that sunscreen use helps protect their skin from later effects (*p* = 0.04). The two groups strongly change their attitudes when considering the best protection, but the LWS group is more convinced that using all types of protection together is best (*p* = 0.04).

**Table 3:** Children’s attitudes in the two groups at T0 and T1.

Children’s attitude (% yes)	T0		T1	
	CTRL ^1^	LWS ^2^	CTRL ^1^	LWS ^2^
**For you, what does it mean to be tan?**				
To be more beautiful?	47.5	46.1	47.4	46.6
To be fashionable or trendy	26.2	24.3	31.8	27.9
To be protected against sunburn?	36.2	37.0	35.4	48.6 ^3^
**When is it necessary to protect yourself from sun exposure?**				
When practicing sports outside?	70.9	74.5	77.6	81.7
At the swimming pool?	58.0	57.8	66.7	68.5
On the beach?	85.2	88.5	89.6	90.2
Walking?	63.9	66.0	69.2	76.7 ^3^
In the mountains?	52.2	48.9	60.0	79.1 ^3^
**Why do you use sunscreen?**				
To avoid sunburn?	56.8	56.9	47.3	51.3
To prolong sun exposure?	8.8	11.4	13.4	15.5
To avoid later skin damage?	14.6	15.0	20.5	27.6 ^3^
Because your parents want you to?	10.2	11.6	19.9	16.5
**What do you think is the best protection against sun risks?**				
Sunscreen use?	10.6	11.8	10.4	7.4
Sunscreen use + hat?	18.2	18.6	9.3	6.1
Sunscreen use + hat + avoid risky hours?	15.3	14.8	15.7	15.3
Sunscreen, hat, avoid risky hour, sunglasses, T-shirt, all together?	46.7	48.1	59.6	67.0 ^3^

^1^ Control classes; ^2^ Experimental classes; ^3^*p* < 0.04.

### 3.4. Results after Summer Holidays

Forty-two school classes participated in this evaluation; 19 from the control group (341 children) and 23 from the LWS group (363 children). The reduction in the number of classes after the holidays is related to administrative moving of teachers and, in some cases, renunciation to follow the programme. This problem was taken into account in the statistical analysis. The characteristics of the children within the two groups compared (age, gender, skin-types) were not different than and were of the same order as the whole initial group (46% of girls, mean age 9.7–9.8, 36% fair eyes, 49–52% fair hair, 56–62% fair skin, 68–71% without freckles, 29–36% easy sunburn). We found the same significant differences for outside activities: The control group performed more often walking or cycling (*p* = 0.027), and swimming-pool (*p* = 0.02).

We also found at T0 the knowledge of this reduced sample was close to the whole group with five significant differences between the compared groups on the 47 questions (four in favour of the LWS group), but only one difference when grouped by headings, also in favour of the LWS group.

After the summer holidays, differences in knowledge between the two groups decreased a little and the differences between T0 and T2 were smaller than between T0 and T1. In the 47 questions at T2, we did not observe a significant difference in the children’s responses in 26 of them, unlike at T1 where we did not observe a significant difference in just 14 of the questions. However, the majority of good responses remained in favour of the LWS group. When grouped by headings, the knowledge of the LWS group was significantly higher in eight of 13 headings.

The rate of good answers at T0 was 58.9% (SD = 10.9%) in the control group *versus* 59.6% (SD = 9.8%) in the LWS group.

**Figure 2:**
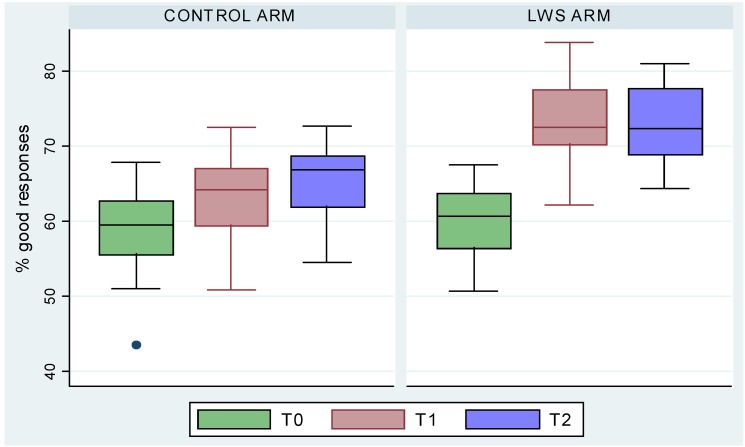
Evolution of score rates of knowledge from T0 to T2 in the two groups.

At T1, this rate was respectively 62.8% (SD = 10.4%) and 73.5% (SD = 10.3%) (*p* < 0.001) and at T2, these rates were 65.2% (SD = 10%) and 72.6% (SD = 9.8%) (*p* < 0.001). Children in the LWS classes were more likely than the control group to state that it was necessary to be protected against the sun, especially when walking in the mountains (Odd Ratio (OR) = 3.01, 95% Confidence Interval (CI) (2.01 *versus* 4.87)), and that the use of sunscreen was a good way to avoid skin damage (OR = 1.54, 95%CI (1.05 *versus* 2.27)) as shown in Figure 2.

Concerning attitudes in this reduced sample, we did not observe any difference at T0 between the two groups. As a whole, they thought: Being tan looks better (46%); that it is necessary to protect themselves from the sun, particularly when practicing sports outside (72.5%) and when they were on the beach (88%); they used sunscreen to avoid sunburn (57.5%) and be protected; and 48% thought it was better to use all types of protection together.

**Figure 3:**
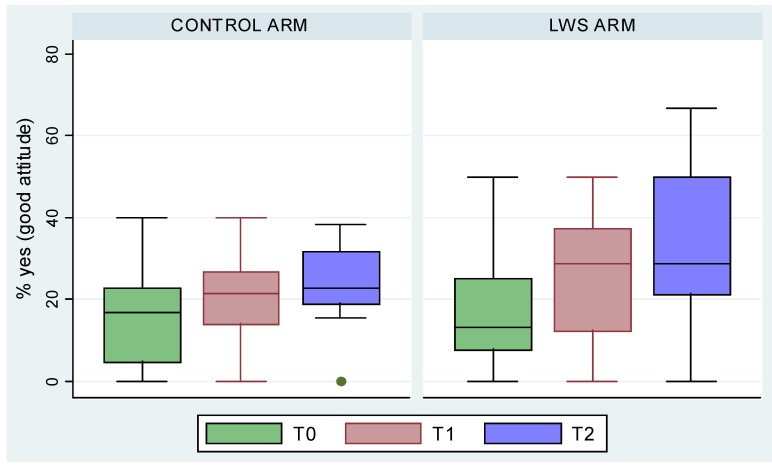
Evolution of attitudes concerning sunscreen from T0 to T2 in the two groups.

When looking at the data at T2, the good attitudes increased in the two groups, but a little bit more in the LWS group; four of the 16 items became significant. The children from LWS classes thought more often (48.6% *versus* 35.4%) that being tan protected them from sunburn (*p* = 0.02) and that sunscreen use helps to protect their skin from later effects (*p* = 0.02). On the contrary, the control group used sunscreen more often because their parents wanted them to and 9% considered it to be the best protection as compared to only 5% in the LWS group (Figure 3).

Finally, we questioned the two groups on their sun behaviours during the last holidays and compared the answers with the data we collected at T0 on their usual sun behaviours (Table 4). Again, the behaviours in the two compared groups were about the same at T0. Of 20 questions, only two items were significantly different: The LWS group was repeating sunscreen application more often during the day and they already had information on the sun at school.

**Table 4:** Declared children’s behaviours before the programme and after the summer holidays (N = 1,004).

Questions and answers (%)	T0		T2	
	CTRL ^1^	LWS ^2^	CTRL ^1^	LWS ^2^
**During the last summer holidays did you experiment:**				
Skin turning red and burning	10.1	20.7	18.5	19.6
Skin burnt and swelling	1.5	0.3	3.5	1.9
Peeled off skin	24.9	23.1	23.2	22.6
Nothing	53.7	53.7	54.8	55.1
**On the beach do you usually used:**				
A T-shirt	54.0	48.7	61.7	59.0
A hat	59.2	66.0	59.0	72.3 ^3^
Sunscreen	89.7	90.5	89.6	91.8
Sun-glasses	55.3	51.7	58.9	55.1
Sunshade	65.8	70.1	64.5	75.2 ^3^
**At the swimming pool do you usually used:**				
A T-shirt	27.6	27.8	27.6	31.4
A hat	29.5	30.5	34.6	39.1
Sunscreen	56.5	55.1	52.4	59.6
Sunglasses	30.2	27.1	23.2	24.7
Sunshade	20.5	22.8	21.0	28.5
Sunscreen various times during the day?	48.9	56.5	57.3	65.1 ^3^
**Where did you find information on sun exposure?**				
At school?	51.0	65.0	58.9	79.1 ^3^
On the web?	19.9	18.5	18.5	22.1
On TV?	44.9	47.9	47.5	40.8
In books	43.1	43.2	38.1	31.4
From your parents?	57.5	59.5	66.9	59.8 ^3^

^1^ Control classes; ^2^ Experimental classes; ^3^*p* < 0.05.

The results at T2 were different (*p* < 0.05) four times out of 20. The LWS group more frequently wore a hat and used more a sunshade when on the beach, they also repeated sunscreen application more often and 79% of them considered their information to have come from school. In the control group, it was the parents who gave information on the sun most often.

### 3.5. Results One Year Later

**Figure 4:**
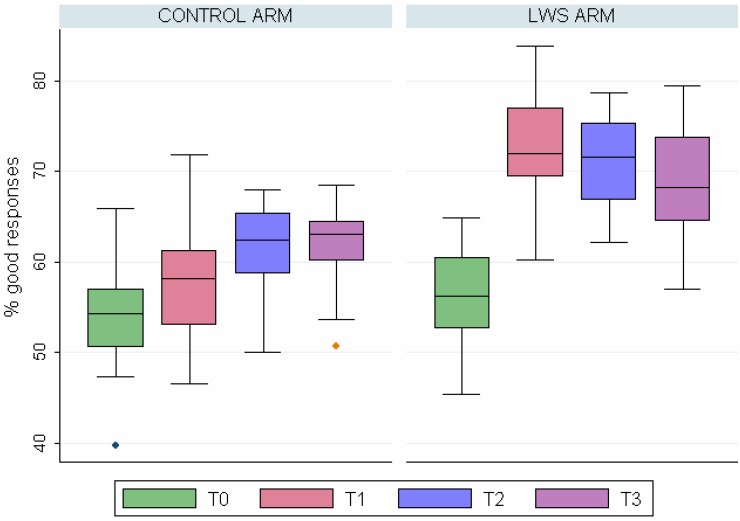
Evolution of score rates of knowledge from T0 to T3 in the two groups.

We were able to collect data in the 33 classes (474 children, 208 from the control and 266 from the LWS group) one year after the completion of the programme. The characteristics of the children within the two compared groups (age, gender, photo-types) remained the same and of the same level as the whole initial group. Difference in knowledge, although less important (62.8% of good answers in the control *versus* 68.5% in the LWS group), was significant (*p* < 0.001) as shown in Figure 4.

When considering protective attitudes, the LWS group still responded significantly better in three items: The children of the LWS group were more convinced of the necessity to protect themselves when practicing water sports (87% *versus* 78%, *p* < 0.04), when being on the beach (96% *versus* 87%, *p* < 0.003), and when being in the mountains (76% *versus* 68%, *p* = 0.05). The declared behaviours were still better in the LWS group only when staying on the beach by using more protective measures, such as wearing a T-shirt, a hat and using a sunshade (Table 5).

**Table 5:** Declared children’s behaviours one year latter (N = 474).

Questions and answers (%)	T0		T3	
	CTRL ^1^	LWS ^2^	CTRL ^1^	LWS ^2^
**On the beach do you usually used:**				
A T-shirt	55.5	48.1	52.2	65.3 ^3^
A hat	60.5	67.1	64.0	73.3 ^3^
Sunscreen	90.1	89.0	86.8	90.4
Sun-glasses	55.3	51.8	68.6	70.2
Sunshade	65.2	71.5	67.2	76.2 ^3^
**At the swimming pool do you usually used:**				
A T-shirt	28.9	25.5	21.2	25.1
A hat	32.8	31.8	32.3	37.2
Sunscreen	54.9	52.1	54.2	59.5
Sunglasses	28.9	26.6	36.5	37.6
Sunshade	21.5	26.1	22.9	27.0
Sunscreen various times during the day?	44.3	56.3 ^3^	59.2	68.0 ^4^

(0 < 0.0574) atter summer holidays, es simple knowledge, could have a greater effect on changing behaviour; ^1^ Control classes; ^2^ Experimental classes; ^3^*p* < 0.10; ^4^*p* = 0.12.

### 3.6. Discussion

The World Health Organization and Centers for Disease Control guidelines [8] recommended developing sun exposure preventive interventions targeting young children because childhood is the time when risk to UV rays is highest. They also recommended targeting childhood interventions at school because children are already present and engaged in the process of learning [8,32]. The “Living with the Sun” guide seems to be a good way to not only increase children’s knowledge, but to obtain modifications of attitudes and behaviour. The guide’s proposed educational activities consists of entertaining scientific games for children as well as a good pedagogical method widely accepted to increase self esteem, which is a good determinant for changing behaviour [9,10,21,33]. Our results clearly confirm that school-based health education programmes can have a positive effect on children knowledge [7]. Furthermore, we also observed in the control group a non-significant increase of the good response score between T0 and T1. We assume that such an increase could be due to the impact of the general environment (schools, parents, television, advertisements, *etc*.) during spring and summer times.

Two systematic reviews [7,27] concluded that education approaches for increasing sun-protection behaviours were somewhat effective when implemented in primary school and in recreational settings. All published studies showed a marked increase in knowledge scores immediately after the programmes concluded and heterogeneous results were obtained for changes in attitude. In our study the differences between the two groups, both on knowledge and attitude concerning sun exposure, decrease with time but are still present after one year as clearly shown in Figure 4.

When considering the problem of positively influencing sun behaviours there are very few interventions that show an effect, moreover the problem of measuring this effect is not easy and hard to reproduce.

The main outcome was a reduced preference for tanning in two studies that included children 9 to 11 years old [17,46]. Our trial was able to clearly demonstrate the efficacy of the LWS programme on modifying attitudes immediately after its conclusion in the schools but also—even more moderate and with a greater variability—after the holidays and one year later. The increase in modified attitudes was related to exercising safe behaviours on the beach.

Some methodological limitations in the statistical analysis may affect the results, for instance, not taking into account dependencies among cluster members [38]. Furthermore, the problem of missing data due to loss of clusters and of children in the long term must be considered. To avoid a significant decrease in statistical power when performing a longitudinal analysis, we chose conservative parameters to estimate the sample size at T0. To estimate the biases due to missing data we used the method proposed by Verbeke and Molenbergs [45]. This enabled us to show that there were no biases created by missing data, especially on the long term results. Finally, the loss of clusters could unbalance the two groups and destroy the randomization. In our study specifically, it was reassuring that when we did a comparison of the characteristics of the clusters, and of the children between the two groups at the different end-points of the trial, that there weren’t any differences in the relevant data influencing sun-exposure.

To evaluate true modifications in behaviours is a real challenge [7,8,19,21,47,48,49]. We were able to get the data of the same children after summer holidays but only by using questionnaires fulfilled by children at school. Our results after the holidays showed an improvement in the sun exposure behaviour in favour of the LWS group (Table 4). However, there was no indication of a difference made on the immediate consequences on skin of inadequate sun exposure as related to children’s responses. We did not undertake external observation of the children’s behaviours or clinical examination by professionals. Such a method, as in other published trials, could create some difficulties for the extrapolation of results.

The maintenance of the LWS curriculum during the following years at schools was not evaluated in our study. Maintenance evaluation has been suggested by many authors [7,8] to reinforce the effect on behaviour, but it has never been evaluated.

## 4. Conclusions

To acquire knowledge that influences new attitudes and supports healthy behaviours is specific to learning in young children. At home, at school and in the street, children progressively discover the outlines of their identity and their place in their social environment. Such experiences are important for the construction of positive self-esteem, choosing their way of life and developing behaviours [50,51].

Our results, based on this theory, showed a significant progression of knowledge and some modification of children’s sun related attitudes and behaviours lasting at least 12 months. In conclusion, the pedagogic approach proposed by the LWS programme was evaluated using a cluster randomised trial adequately analysed, the study was able to confirm previous published results on acquisition of knowledge and to add new arguments concerning change in attitude and short term behaviour particularly when being on the beach. It seems that primary school is a favourable site and period in time to communicate messages about excessive exposure to the sun. Educational methods, such as using pragmatic and funny tools delivered by trained teachers over a long period of time, could also be a part of the LWS programme’s success. From this research it would seem school-based health education to promote sun safety behaviour is most effective when it is provided consistently and included periodically in every grade of the school curriculum from pre-kindergarten to secondary school (18). Such policies still have to be developed in French schools.

Finally, interventions in schools should be part of larger programmes involving parents and communities and they should rely not only on one cognitive approach, but aim at changing children’s environment and behaviours through many.

## References

[1] Rosso S., Zanetti R., Pipione M., Sancho-Garnier H. (1998). Parallel risk assessment of melanoma and basal cell carcinoma: Skin characteristics and sun-exposure. Melanoma Res..

[2] Taylor H.R., West S.K., Rosenthal F.S., Munoz B., Newland H.S., Abbey H., Emmet E.A. (1988). Effect of ultraviolet radiation on cataract formation. N. Engl. J. Med..

[3] Younf R.W. (1988). Solar radiation and age-related macular degeneration. Surv. Ophtalmol..

[4] Dennis L.K., van Beek M.J., Beane Freeman L.E., Smith B.J., Dawson D.V., Coughlin J.A. (2008). Sunburns and risk for cutaneous melanoma: Does age matter? A comprehensive meta-analysis. Ann. Epidemiol..

[5] Linos E., Swetter S.M., Cockburn M.G., Colditz G.A., Clarke C.A. (2009). Increasing burden of melanoma in the United States. J. Invest. Dermatol..

[6] Institut National de Veille Sanitaire. http://www.invs.sante.fr/surveillance/cancers/estimations_cancers/donnees_localisation.

[7] Stoebner-Delbarre A., Defez C., Borrel E., Sancho-Garnier H., Guillot B., Groupe EPI-CES (2005). Prevention of skin cancer programs: Analysis of the impact of randomized trials. Ann. Dermatol. Venereol..

[8] (2011). Behavioral Counseling to Prevent Skin Cancer: Systematic Evidence Review to Update the 2003 US Preventive Services Task Force Recommendation; Report number 11-05152-EF-1.

[9] Sandrin-Berthon B. (1997). Apprendre à l’Ecole.

[10] Mermelstein R.J., Riesenberg L.A. (1992). Changing knowledge and attitudes about skin cancer risk factors in adolescents. Health Psychol..

[11] Hugues B.R., Altman D.G., Newton J.A. (1993). Melanoma and skin cancer: Evaluation of a health education programme for secondary schools. Br. J. Dermatol..

[12] Buller D.B., Buller M.K., Beach B., Ertl G. (1996). Sunny days, healthy ways” evaluation of a skin cancer prevention curriculum for elementary school-aged children. J. Am. Acad. Dermatol..

[13] Buller D.B., Hall J.R., Powers P.J., Ellsworth R., Beach B.H., Frank C.A., Maloy J.A., Buller M.K. (1999). Evaluation of the “Sunny Days, Healthy Ways” sun safety CD-Rom program for children in grades 4 and 5. Cancer Prev. Control..

[14] Lowe J.B., Balanda K.P., Stanton W.R., Gillespie A. (1999). Evaluation of a three-year school-based intervention to increase adolescent sun protection. Health Educ. Behav..

[15] Milne E., English D.R., Cross D., Corti B., Costa C., Johnston R. (1999). Evaluation of an intervention to reduce sun exposure in children. Am. J. Epidemiol..

[16] Hornung R.L., Lennon P.A., Garrett J.M., DeVellis R.F., Weinberg P.D., Strecher V.J. (2000). Interactive computer technology for skin cancer prevention targeting children. Am. J. Prev. Med..

[17] Barankin B., Liu K., Howard J., Guenther L. (2001). Effects of a sun protection program targeting elementary school children and their parents. J. Cutan. Med. Surg..

[18] Glanz K., Geller A.C., Shigaki D., Maddock J.E., Isnec M.R. (2002). A randomized trial of skin cancer prevention in aquatics settings: The Pool Cool program. Health Psychol..

[19] Naldi L., Chatenoud L., Bertuccio P., Zinetti C., Di Landro A., Scotti L., La Vecchia C., Oncology Cooperative Group of the Italian Group for Epidemiologic Research in Dermatology (GISED). (2007). Improving sun protection behaviour among children: Results of a cluster-randomized trial in Italian elementary schools. The “SoleSi-SoleNo-GISED” project. J. Invest. Dermatol..

[20] Olson A.L., Gaffney C., Starr P., Gibson J.J., Cole B.F., Dietrich A.J. (2007). Sunsafe in the middle school years: A community wide intervention to change early adolescent sun protection. Pediatrics.

[21] Hunter S., Love-Jacson K., Abdulla R., Zhu W., Lee J.H., Wells K.J., Roetzheim R. (2010). Sun protection at elementary schools: A cluster randomized trial. J. Natl. Cancer Inst..

[22] Roetzheim R.G., Love-Jacson K.M., Hunter S.G., Lee J.H., Chen R., Abdulla R., Wells K.J. (2011). A cluster randomized trial of sun protection at elementary schools. Results from year 2. Am. J. Prev. Med..

[23] Milne E., Johnston R., Cross D., Giles-Corti B., English D.R. (2002). Effect of a school-based sun-protection intervention in the development of melanocytic nevi in children. Am. J. Epidemiol..

[24] English D.R., Milne E., Jacoby P., Giles-Corti B., Cross D., Johnston R. (2005). The effect of a school-based sun protection intervention in the development of melanocytic nevi in children: 6 year follow-up. Cancer Epidemiol. Biomarkers Prev..

[25] Bauer J., Buttner P., Wiecker T.S., Luther H., Garbe C. (2005). Interventional study in 1232 young German children to prevent the development of melanocytic nevi failed to change sun exposure and sun protective behavior. Int. J. Cancer.

[26] Harrison S.L., Buettner P.G., MacLennan R. (2005). The North Queensland “Sun Safe Clothing” study: Design and baseline results of a randomized trial to determine the effectiveness of sun-protective clothing in preventing melanocytic nevi. Am. J. Epidemiol..

[27] Saraiya M., Glanz K., Briss P.A., Nichols P., White C., Das D., Jay Smith S., Tannor B., Hutchinson A.B., Wilson K.M. (2004). Interventions to prevent skin cancer by reducing exposure to ultraviolet radiations: A systematic review. Am. J. Prev. Med..

[28] Wilgenbus D., Cesarini P., Bense D. (2005). Vivre avec le Soleil, Guide de L’Enseignant.

[29] Quéreux G., Nguyen J.M., Volteau C., Dréno B. (2009). Prospective trial on a school-based skin cancer prevention project. Eur. J. Cancer Prev..

[30] Fitzpatrick T.B. (1988). The validity and practicability of sun-reactive skin types I through to VI. Arch. Dermatol..

[31] Defez C., Stoebner-Delbarre A., Sancho-Garnier H. (2002). La prévention des cancers cutanés: Résultats de quelques programmes. Oncologie.

[32] Peters L., Paulussen T. (1997). School Health Promotion and Cancer Prevention. A Review of International Effect Research on Skin Cancer Prevention.

[33] Conner M., Norman P. (1995). Predicting Health Behavior: Research and Practice with Social Cognition Model.

[34] Fishbein M., Ajtzen I. (1975). Belief, Attitude, Intention, and Behavior.

[35] Ajtzen I., Kuhl J., Beckmann J. (1985). From intention to actions: A Theory of Planned Behavior. Action-Control: From Cognition to Behavior.

[36] Buller D.B., Reynolds K.D., Yaroch A., Cutter G.R., Hines J.M., Geno C.R., Maloy J.A., Brown M., Woodall W.J., Grandpre J. (2006). Effects of the “Sunny Days Healthy Way” curriculum on students in grades 6 to 8. Am. J. Prev. Med..

[37] Donner A., Klar N. (2000). Design and Analysis of Cluster Randomization Trials in Health Research.

[38] Murray D.M., Varnell S.P., Blitstein J.L. (2004). Design and analysis of group-randomized trials: A review of recent methodological developments. Am. J. PublicHealth.

[39] Zanetti R., Rosso S., Martinez C., Navarro C., Schraub S., Sancho-Garnier H., Franceschi S., Gafa L., Perea E., Tormo M.J. (1996). The multicentre south European study “Helios” I: Skin characteristics and sunburns in basal cell and squamous cell carcinomas of the skin. Br. J. Cancer.

[40] Rosso S., Zanetti R., Martinez C., Tormo M.J., Schraub S., Sancho-Garnier H., Franceschi S., Gafa L., Perea E., Navarro C. (1996). The multicentre south European study “Helios” II: Different sun exposure patterns in the aetiology of basal cell and squamous cell carcinomas of the skin. Br. J. Cancer.

[41] Sancho-Garnier H., Dubertret L. (2006). Prévention des Cancers Cutanés: Connaissance, Attitudes et Comportements. Soleil et Santé (Rapport de l’Académie Nationale de Médecine).

[42] Klar N., Darlington G. (2004). Methods for modelling in cluster randomization trials. Stat. Med..

[43] Benjamini Y., Hochberg Y. (1995). Controlling the false discovery rate: A practical and powerful approach to multiple testing. J. R. Statist. Soc. Ser. B.

[44] Pereira B., Sancho-Garnier H., Kramar A. Methodological Developments of Cluster Randomised Trials (CRT)—How to Analyse a CRT with Repeated Measures?. Proceedings of the International Congress of Epidemiology IEA-EPI.

[45] Verbeke G., Molenbergs M. (2000). Linear Mixed Models for Longitudinal Data.

[46] Daurès J.P., Sancho-Garnier H., Pourrin-Bourdonneau C., Fissier M., Arnaud C., Grabbar S., Vergnes C., Picot E., Meynadier J. (1995). Etude des facteurs démographiques, environnementaux et congénitaux de développement des naevi chez l’enfant entre 3 et 15 ans. Premiers résultats. Rev. Epidém. et Santé Publ..

[47] Abar B.W., Turrisi R., Hillhouse J., Loken E., Stapleton J., Gunn H. (2010). Preventing skin cancer in college females: Heterogeneous effects over time health. Psychology.

[48] Escoffery C., Glanz K., Hall D., Elliot T. (2009). A multi-method process evaluation for a skin cancer prevention diffusion trial. Eval. Health Prof..

[49] Buller D.B., Reynolds K.D., Ashley J.L., Buller M.K., Kane I.L., Stabe C.L., Massie K.L., Liu X., Cutter G.R. (2011). Motivating public school districts to adopt sun protection policies: A randomized controlled trial. Am. J. Prev. Med..

[50] Azorin J.C., Stoebner-Delbarre A., Sancho-Garnier H. (2007). L’Education Promotricede Santé des Enfants de 3 à 11 ans. Au Delà de L’information la Prévention.

[51] Slama K. (2004). Health Behavior and Change. Evidence-Based Cancer Prevention: Strategies for NGOs: A UICC Handbook for Europe.

